# Supplementary material for: Coexistence of structural and magnetic phases in van der Waals magnet CrI3
Source: Nat Commun. 2021 Nov 1;12:6265. doi: 10.1038/s41467-021-26342-4 (PMC8560937; doi:10.1038/s41467-021-26342-4)
Supplement: Supplementary file 1 — Supplementary Information [file 41467_2021_26342_MOESM1_ESM.pdf]

Supplementary Information:
Coexistence of structural and magnetic phases in van der Waals magnet CrI_3

Jaume Meseguer-Sánchez[1], Catalin Popescu[2], José Luis García Muñoz[3], Hubertus Luetkens[4], Grigol Taniashvili[5], Efrén Navarro-Moratalla[1,†], Zurab Guguchia[4,†], Elton J. G. Santos[6,7,†]

[1] *Instituto de Ciencia Molecular, Universitat de Valéncia, Calle Catedrático José Beltrán Martínez 2, 46980, Paterna, Spain*

[2] *CELLS-ALBA Synchrotron, 08290 Cerdanyola del Vallés, Catalonia, Spain*

[3] *Institut de Ciència de Materials de Barcelona (ICMAB), CSIC, 08193 Bellaterra, Catalunya, Spain.*

[4] *Laboratory for Muon Spin Spectroscopy, Paul Scherrer Institute, CH-5232 Villigen PSI, Switzerland*

[5] *Department of Physics, Tbilisi State University, Chavchavadze 3, GE-0128 Tbilisi, Georgia*

[6] *Institute for Condensed Matter Physics and Complex Systems, School of Physics and Astronomy, The University of Edinburgh, EH9 3FD, UK*

[7] *Higgs Centre for Theoretical Physics, The University of Edinburgh, EH9 3FD, UK*

[†] *Correspondences to: efren.navarro@me.com, zurab.guguchia@psi.ch, esantos@ed.ac.uk*

Contents

1 CrI_3 bulk crystal growth **2**

2 μ-SR experiment and analysis 4

3 SQUID magnetometry 8

4 Synchrotron X-ray diffraction measurements 9

5 Additional analysis on X-ray data 10

6 Supplementary References 13

1 CrI_3 bulk crystal growth

Chromium triiodide crystals were grown using the chemical vapour transport technique. Chromium powder (99.5% Sigma-Aldrich) and anhydrous iodine beads (99.999% Sigma-Aldrich) were mixed in a 1:3 ratio in an argon atmosphere inside a glovebox. 972 mg of the mixture were loaded into a silica ampoule with a length, inner diameter and outer diameter of 500 mm, 15 mm and 16 mm respectively (Supplementary Figure 1**a**). The ampoule was extracted from the glovebox with a ball valve covering the open end to prevent air exposure and then it was immediately was evacuated using a turbo-molecular pump down to 6×10^{-6}. Once the pressure stabilised, the closed end was dipped in liquid nitrogen to prevent the sublimation of the iodide beads. The ampoule was then flame sealed and introduced into a three-zone furnace. An initial inverted gradient step was used to minimize nucleation sites in the growth zone. The gradient was then inverted so that the source

zone would remain at 650 ^oC, the middle growth zone at 550 ^oC and the third zone at 600 ^oC for 3 days. Crystal growth takes place in the centre and source zones. The single crystals and the microcrystalline powder were both extracted from the ampoule and stored in an argon atmosphere inside the glovebox to prevent oxidation and hydration. A sample of the bigger single crystals was ground into a powder using a razor blade. X-Rays diffraction analysis was performed on samples of the powder and single crystalline CrI_3 by loading the material into a capillary and flame-sealing it inside the glove box. The powder pattern of both samples were consistent with the monoclinic $AlCl_3$-type structure (C2/m) reported for CrI_3 (Supplementary Figure 1**b**).

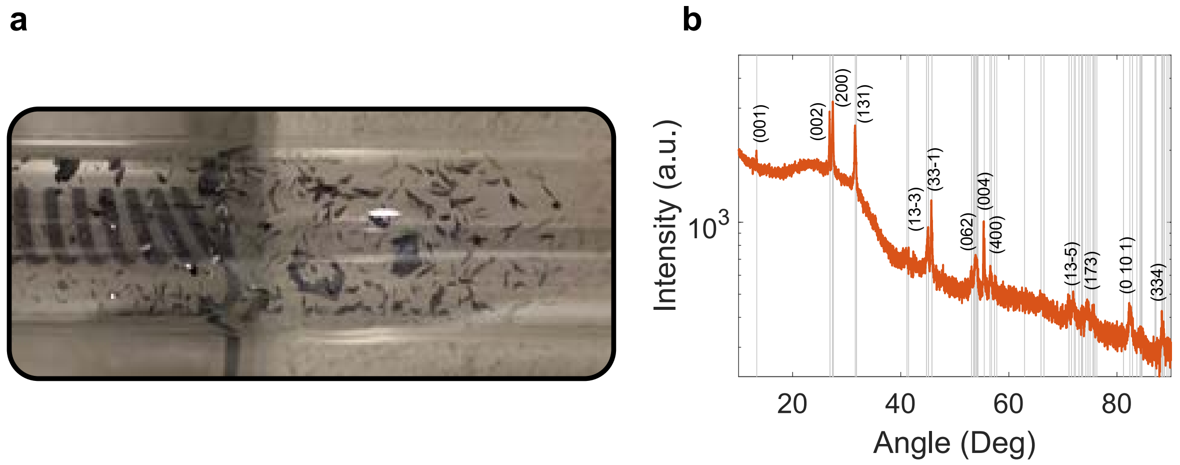

Supplementary Figure 1: **CrI_3 samples. a,** Picture of the ampoule used for sample fabrication. **b,** X-Ray diffraction pattern of the CrI_3 microcrystalline powder studied in the μ-SR experiment. The most prominent peaks of the orange trace (experiment) have been labelled (Miller indices) and marked (grey lines) with peaks positions of the monoclinic (room temperature) crystalline phase of CrI_3.

2 μ-SR experiment and analysis

The μ-SR method is based on the observation of the time evolution of the spin polarization $\vec{P}(t)$ of the muon ensemble. In μ-SR experiments an intense beam (p_μ = 29 MeV/c) of 100 % spin-polarized muons is stopped in the sample. Currently available instruments allow essentially a background free μ-SR measurement at ambient conditions [1]. The positively charged muons thermalize in the sample at interstitial lattice sites, where they act as magnetic microprobes. In a magnetic material the muons spin precess in the local field B_μ at the muon site with the Larmor frequency $\nu_\mu = \gamma_\mu/(2\pi)B_\mu$ (muon gyromagnetic ratio $\gamma_\mu/(2\pi)$ = 135.5 MHz T^{-1}). The muons μ^+ implanted into the sample will decay after a mean life time of τ_μ = 2.2 μs, emitting a fast positron e^+ preferentially along their spin direction. Various detectors placed around the sample track the incoming μ^+ and the outgoing e^+. When the muon detector records the arrival of a μ in the specimen, the electronic clock starts. The clock is stopped when the decay positron e^+ is registered in one of the e^+ detectors, and the measured time interval is stored in a histogramming memory. In this way a positron-count versus time histogram is formed. A muon decay event requires that within a certain time interval after a μ^+ has stopped in the sample a e^+ is detected. This time interval extends usually over several muon lifetimes (*e.g.* 10μs).

The GPS (πM3 beamline) μ-SR instrument at the Paul Scherrer Institute, Switzerland, was used to study the CrI_3 sample. The specimen was mounted in a He gas-flow cryostat and the temperature was varied between 5 and 200 K.

Analysis of Weak TF-μSR data The weak TF asymmetry spectra were analyzed [2,3] using the function:

$$A_S(t) = A_p \exp(-\lambda t)\cos(\omega t + \phi). \tag{1}$$

where t is the time after muon implantation, $A(t)$ is the time-dependent asymmetry, A_p is the amplitude of the oscillating component (related to the paramagnetic volume fraction), λ is an exponential damping rate due to paramagnetic spin fluctuations and/or nuclear dipolar moments, $\omega = 2\pi\nu_\mu$ is the Larmor precession frequency set by the strength of the transverse magnetic field, and ϕ is a phase offset. The zero for $A(t)$ was allowed to vary for each temperature to deal with the asymmetry baseline shift occurring for magnetically ordered samples. From these refinements, the magnetically ordered volume fraction at each temperature T was obtained by V_M = 1 - $A_p(T)/A_p(T_{max})$, where $A_p(T_{max})$ is the amplitude in the paramagnetic phase at high temperature.

Analysis of ZF μ-SR data In the paramagnetic state, the μ-SR spectra are described by the combination of Lorentzian and Gaussian Kubo-Toyabe depolarization function:

$$A_{\mathrm{ZF,PM}}(t) = (1 - V_M)A_0 \left(\frac{1}{3} + \frac{2}{3}\left(1 - (\sigma t)^2 - \Lambda t\right) e^{-\frac{1}{2}(\sigma t)^2 - \Lambda t} \right) \tag{2}$$

Here, the depolarisation rates σ and Λ are due to the nuclear dipole moments and randomly oriented diluted local electronic moments, respectively. A_0 is the initial asymmetry. V_{PM} = 1 - V_M is the paramagnetic fraction, which acquires maximum value of 1 above 60 K.

In the ordered state, the response of the sample consists of three magnetic components, depending on temperature:

$$A_{\mathrm{ZF,M}}(t) = V_M A_0 \omega_1 \left(f_{T1} \cos(\gamma_\mu B_{\mu 1} t + \phi) e^{-\lambda_{T1} t} + f_{L1} e^{-\lambda_{L1} t} \right) + \cdots \tag{3}$$

$$V_M A_0 \omega_2 \left(f_{T2} \cos(\gamma_\mu B_{\mu 2} t + \phi) e^{-\lambda_{T2} t} + f_{L2} e^{-\lambda_{L2} t} \right) + \cdots \tag{4}$$

$$V_M A_0 \omega_3 \left(f_{T3} e^{-\lambda_{T3} t} + f_{L3} e^{-\lambda_{L3} t} \right) \tag{5}$$

Here, ω_1, ω_2, and ω_3 are the relative fractions of the magnetic components. The first and second terms describe the high and low frequency magnetic components, respectively. The components are characterised by an oscillating "transverse" component and a slowly relaxing "longitudinal" component. The longitudinal component arises due to muons experiencing local field

components which are parallel to the initial muon spin polarization. In polycrystalline samples with randomly oriented fields the orientational averaging results in a so-called "one-third tail" with $f_L = \frac{1}{3}$ ($f_T = \frac{2}{3}$). For single crystals, f_L varies between zero and unity as the orientation between field and polarization changes from being perpendicular to parallel. λ_T and λ_L are the transverse and longitudinal depolarisation rates of the μ-SR signals, respectively. $B_{\mu 1}$ and $B_{\mu 2}$ are the internal magnetic fields at the muon site, corresponding to high and low frequency components, respectively. The third term describes the strongly damped magnetic component, which occurs below 30 K only. We note that it is not possible to separate λ_{L3} from λ_{L1} and λ_{L2} and thus it is fixed to 0 in the analysis.

3 SQUID magnetometry

Magnetization curves and zero-field-cooled/field-cooled susceptibility sweeps were carried out in a SQUID magnetomoter (Quantum Design MPMS-XL-7) on single crystals of CrI_3 were the relative orientation of the basal plane of the sample with the external magnetic field (both AC and DC) is controlled. In order to measure the magnetization of CrI_3 in the presence of in-plane and out-of-plane external magnetic fields, two different samples were prepared. For the out-of-plane orientation, various single crystals were stacked one on top of the other and placed onto the surface on the end of a polyethylene rod. This was carefully placed inside a silica tube with the crystals facing upwards and another polyethylene rod placed on top trapping the crystal in between. For the in-plane orientation, a polyethylene rod was cut longitudinally and a single crystal was glued to the flat using Apiezon-N grease. These preparations were performed in an argon atmosphere to avoid crystal degradation. The samples were then inserted inside an outer silica tube and flame sealed at a pressure lower than 10^{-3} mbar.

4 Synchrotron X-ray diffraction measurements

Synchrotron X-ray powder diffraction (SXRPD) measurements were performed on the BL04-MSPD beam-line of the ALBA Synchrotron Light Facility (Barcelona, Spain) using the multi-crystal analyser MAD detector system. The wavelength, $\lambda = 0.38670(3)$ Å, was determined by measuring a NIST standard silicon. This energy (32 KeV) was selected in order to avoid the fluorescence from iodine ions (33 KeV). The sample was loaded in a borosilicate glass capillary of 0.3 mm diameter to keep the absorption below 1, and was kept spinning during data acquisition. Patterns between 10 and 300 K were collected using a Dynaflow liquid He cryostat. Measurements were performed at fix temperatures in warming conditions, after a first cooling down to 10 K.

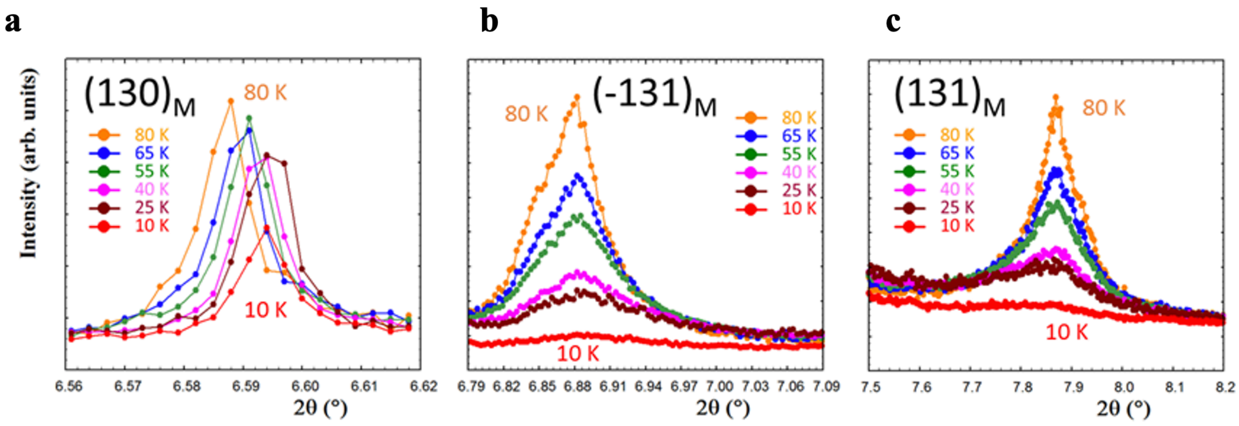

Supplementary Figure 2: **Temperature dependent X-ray diffraction. a-c,** Temperature evolution of selected monoclinic reflections below 80 K (MSPD Alba). Panels show the persistence of the monoclinic phase even at low temperatures. Notice the wide Lorentzian shape of the (±1L1) family of planes.

5 Additional analysis on X-ray data

To illustrate the coupling between structures and magnetic phases in a different way, Supplementary Figures S3−4 show the SXRPD intensity profiles of some R-peaks at the following selected temperatures: 80, 65, 55, 40, 25 and 10 K. In Supplementary Figure S3 we compare the thermal evolution of $(003)_R$ and $(116)_R$ profiles. Firstly, a sudden increase in the intensity is apparent in both below 55 K. Secondly, and more interestingly, there is a remarkable intensity drop in $(003)_R$ below 25 K, which concurs with a large increase of the intensity of $(116)_R$. These small but reliable changes strongly suggest a coupling between the structure and the magnetic transitions in CrI_3.

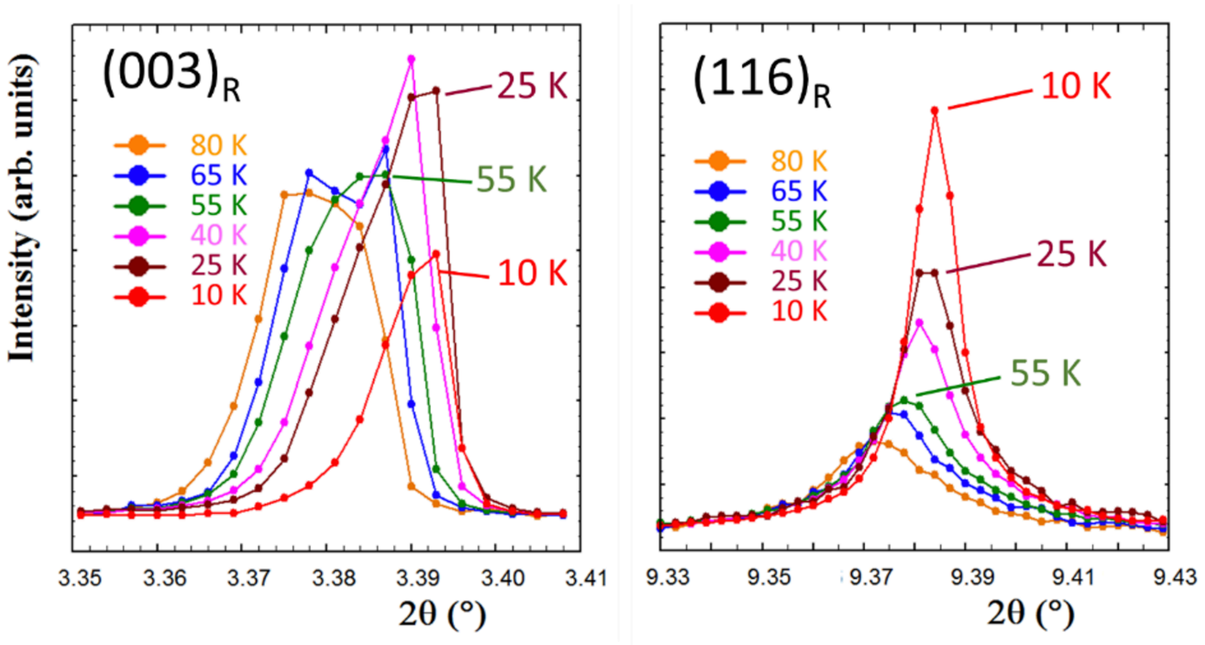

Supplementary Figure 3: **Temperature dependent X-ray diffraction.** Anomalous temperature evolution close to the magnetic transition temperatures of the $(003)_R$ (left) and $(116)_R$ (right) rhombohedral peaks (MSPD Alba). Left: Sudden $(003)_R$ intensity increase below 55 K and sharp drop below 25 K. Right: Prominent $(116)_R$ intensity increase below 55 K and additional increase below 25 K, concurrent with the drop in $(003)_R$.

Similar intensity changes were observed in most of the R-peaks. As a further example, Supplementary Figure S4 shows the changes at low temperatures in the intensity of six additional diffraction peaks. A marked intensity raise below 55 K is apparent in $(327)_R$, $(416)_R$, $(238)_R$, $(051)_R$. The highest intensity is reached at 25 K for $(101)_R$, $(300)_R$, $(238)_R$, $(051)_R$ whereas a substantial intensity drop occurs at 10 K. The evolution of these peaks is reminiscent of that in Supplementary Figure S3, and it is in contrast with reflections $(327)_R$ or $(416)_R$ in which (mirroring the peak $(116)_R$ in Fig. S3) keeps growing also below 25 K. Therefore, this anomalous evolution is consistent with small structural changes causing certain relative intensity variations within the low-temperature range.

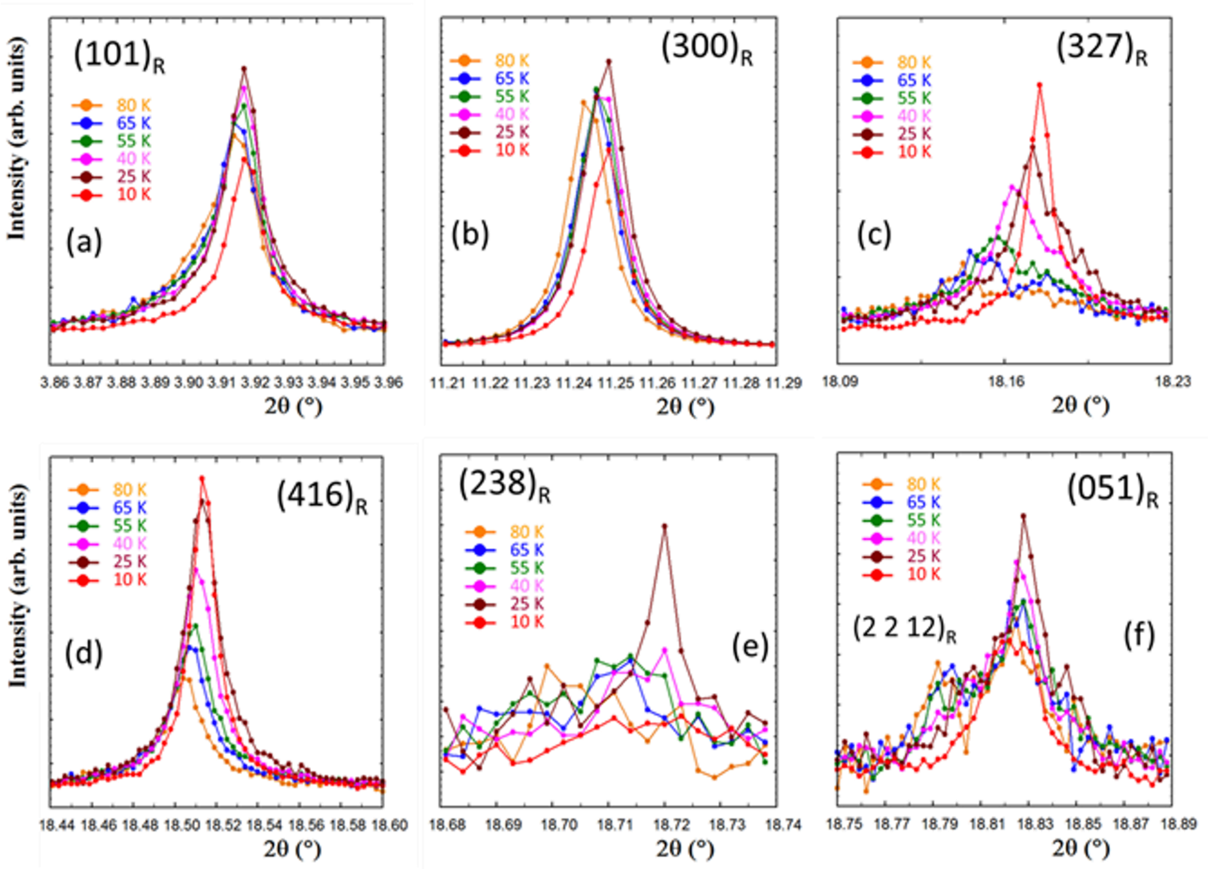

Supplementary Figure 4: **Temperature dependent X-ray diffraction.** Temperature evolution of some rhombohedral peaks of CrI3 (MSPD Alba) showing different trends in the temperature dependence below 80 K.

6 Supplementary References

1. Amato, A. *et al.* The new versatile general purpose surface muon instrument. *Review of Scientific Instruments* **88**, 093301 (2017). URL `https://doi.org/10.1063/1.4986045`.

2. Suter, A. & Wojek, B. Musrfit: A free platform-independent framework for musr data analysis. *Physics Procedia* **30**, 69 – 73 (2012). URL `http://www.sciencedirect.com/science/article/pii/S187538921201228X`.

3. Guguchia, Z. *et al.* Tunable anomalous hall conductivity through volume-wise magnetic competition in a topological kagome magnet. *Nature Communications* **11**, 559 (2020). URL `https://doi.org/10.1038/s41467-020-14325-w`.
